# A Mysterious Case of Left Shoulder Pain in AIDS: A Case Report and Literature Review

**DOI:** 10.7759/cureus.38884

**Published:** 2023-05-11

**Authors:** Kendall Johnson, Henrik Ghantarchyan, Brandon Johnston, Veerpal Sond, Dan Vo

**Affiliations:** 1 Internal Medicine, California University of Science and Medicine, Colton, USA; 2 Internal Medicine, Arrowhead Regional Medical Center, Colton, USA

**Keywords:** hiv, shoulder pain, neisseria gonorrhea, disseminated gonorrhea, aids

## Abstract

Neisseria (N.) gonorrhea is a gram-negative diplococcus and one of the most commonly reported sexually transmitted infections (STIs) in the United States. Disseminated gonococcal infection is a rare but serious complication of N. gonorrhoeae infection that can result in arthritis-dermatitis syndrome or purulent gonococcal arthritis. Co-infection with human immunodeficiency virus (HIV) has been shown to reduce the efficacy of complement recruitment, which may lead to an increased risk of disseminated gonococcal spread. We present a case of a 41-year-old male with concomitant HIV-gonorrhea infection complicated by rare chronic subacute septic arthritis localized to the left shoulder. The patient had a history of HIV, hypertension, and diabetes, and presented with symptoms, including diarrhea, oral thrush, body aches, and fevers. During his hospitalization, the patient developed increasing left shoulder pain, and imaging and joint aspiration revealed N. gonorrhoeae as the causative agent. The patient was treated with appropriate antibiotics and showed improvement. This case highlights the importance of considering disseminated gonococcal infection as a potential complication of N. gonorrhoeae infection, particularly in patients with concomitant HIV infection, and the need for prompt diagnosis and appropriate treatment to prevent complications.

## Introduction

Human immunodeficiency virus (HIV) is a retroviral infection contracted through contact with blood products, sexual intercourse, IV drug use, or nosocomial exposure. HIV selectively targets CD4+ cells, ultimately leading to severe immunocompromise without treatment. HIV progresses to acquired immunodeficiency syndrome (AIDS) when a patient’s CD4+ cell count is below 200 cells/mm^3^ of blood or when a patient presents with any of a number of AIDS-defining illnesses (e.g. Kaposi sarcoma, HIV-related encephalopathy, Pneumocystis jirovecii) regardless of CD4+ count. There is an increased risk of opportunistic infections in AIDS patients due to HIV-induced leukopenia [1].

History of an STI infection is a well-established risk factor for HIV infection; previous studies have estimated rates of HIV-gonorrhea coinfection at 9.5% [2]. While HIV and gonorrhea are known to spread through contact with blood-based fluids and mucous membranes, respectively, gonorrhea has also demonstrated a propensity to spread hematogenously to cause localized septic arthritides, most commonly in the knees [3]. We report the disease course of a patient with HIV-gonorrhea coinfection complicated by rare septic arthritis localized to the left shoulder.

## Case presentation

A 41-year-old Hispanic male with a history of HIV for 15 years, hypertension, and diabetes presented to the ED with worsening diarrhea and new-onset oral thrush. The patient reported persistent non-bloody diarrhea for more than four weeks, endorsing approximately 10 episodes per day. The patient also complained of nausea, vomiting, anorexia with trouble swallowing, body aches, malaise, night sweats, double vision, and episodic fevers. He was diagnosed with HIV in 2007 and was prescribed bictegravir-emtricitabine-tenofovir and trimethoprim-sulfamethoxazole and was adherent to medication up until two months prior to admission due to the medication cost. He also reports having meningitis and syphilis in the past, both of which were treated. The patient had a history of multiple male sexual partners and inconsistent condom use. He admitted to sexual intercourse in the months prior to arrival. He denied any history of smoking, drinking, or intravenous (IV) drug use. No pertinent family or surgical history was reported. He denied a penile discharge, ulcers, skin rashes, dysuria, headaches, shortness of breath, cough, or sick contacts. 

On arrival at the ED, the patient was afebrile and hemodynamically stable. Physical examination was remarkable for cachexia with evidence of temporal wasting. Oral thrush was observed with thick, white plaque extending to the posterior pharynx. There was mild tenderness to palpation throughout all four quadrants of the abdomen. Upper extremities had decreased range of motion with no observed rashes or skin lesions.

His CD4 and viral load were obtained (Table 1). A venereal disease research laboratory (VDRL) test for syphilis was reactive with a titer of 1:256. During the hospital course, prophylactic trimethoprim-sulfamethoxazole was started because of the low CD4 count. He was treated for esophagitis. Given that he endorsed previous syphilis treatment a year ago, we obtained a VDRL 1:256. There was a high suspicion of re-infection. A CSF VDRL was obtained and resulted as negative. Since we were unable to confirm prior syphilis treatment through our public health records, we treated for late latent syphilis. We obtained an acid-fast bacilli (AFB) smear of the sputum, which was positive for mycobacterium avium complex (MAC). He has upcoming appointments to start treatment for MAC. Bictegravir-emtricitabine-tenofovir alafenamide was started during this admission as well.

**Table 1:** Significant HIV labs Legend: *μL = microliter, g = gram, dL = deciliter, mEq = milliequivalent, mmol = millimole, mg = milligram, mL = milliliter

Blood Test Results	Patient Value	Reference Range
CD4 count (cells/uL)	78	490-1740
HIV-1 viral load RNA, PCR (copies / mL)	129,000	<20

**Figure 1:**
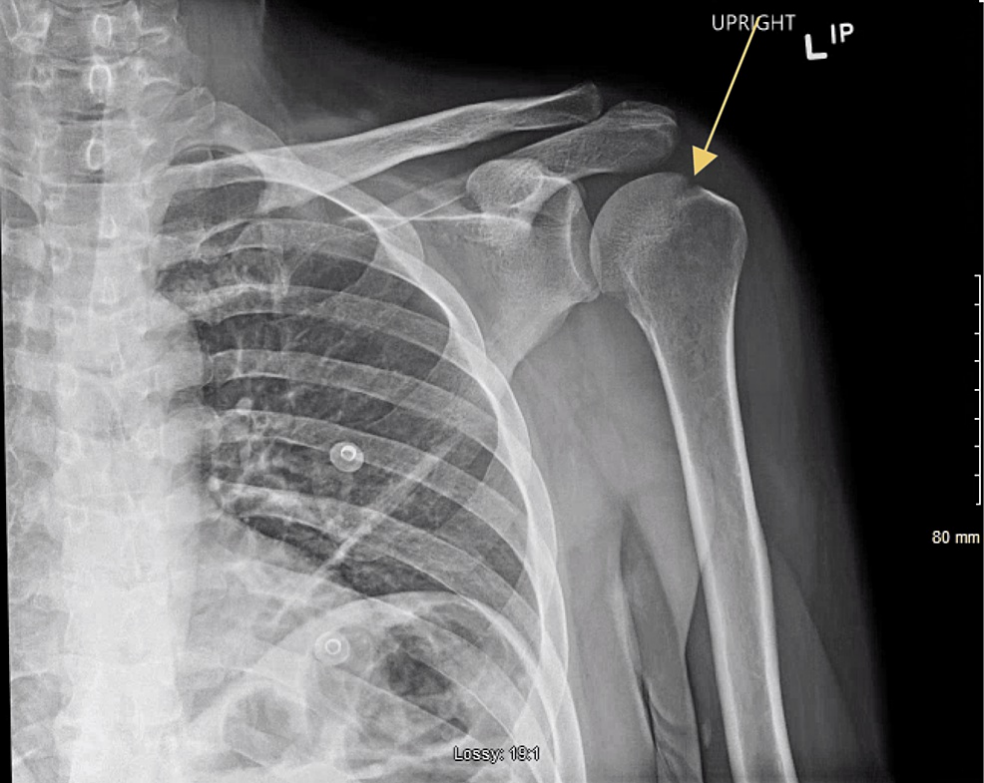
Radiographic findings of the left shoulder Description: Anterior-posterior shoulder radiograph demonstrating joint effusion with focal erosion of the left humeral head (yellow arrow)

**Figure 2:**
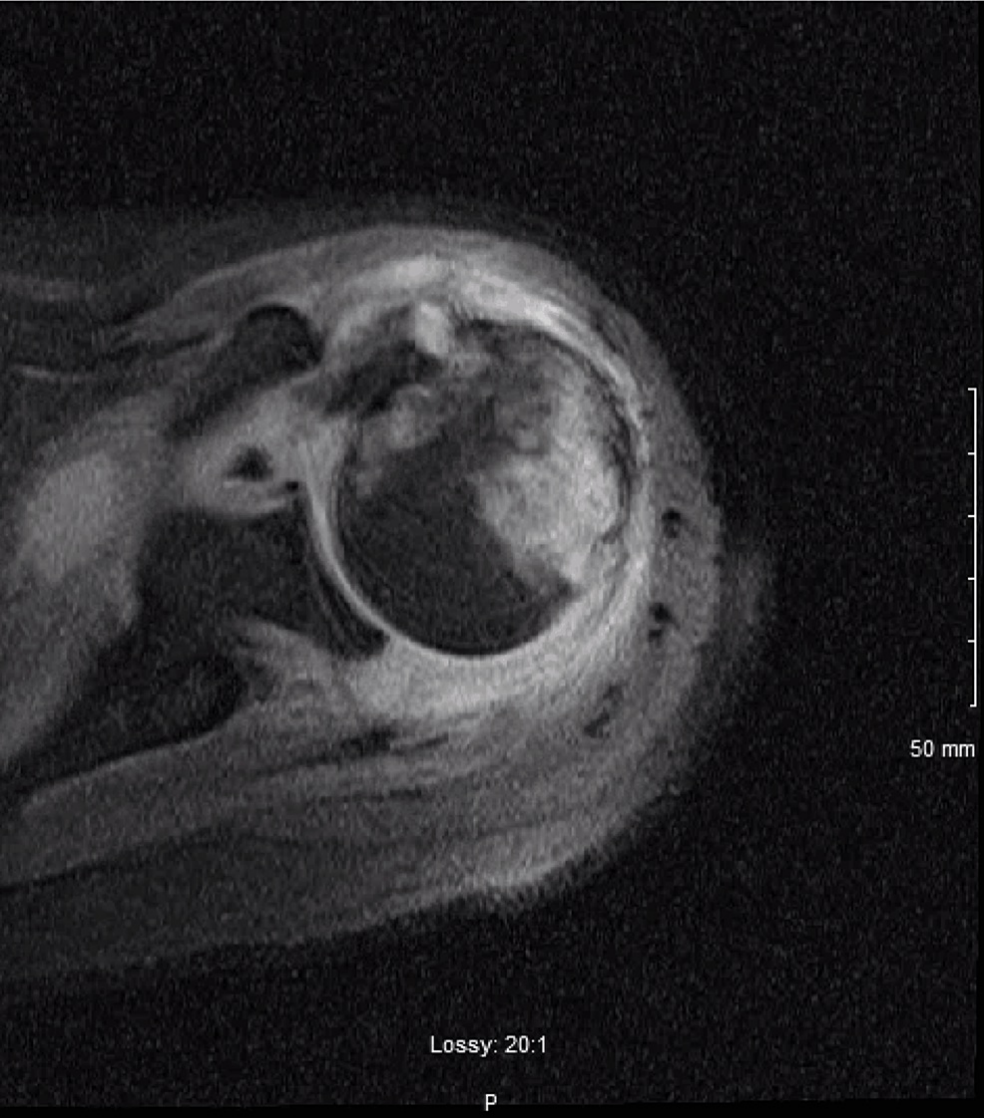
Magnetic resonance imaging (MRI) of the left shoulder Description: Non-contrast magnetic resonance imaging of the left shoulder demonstrating inflammation and edema of the joint space

**Figure 3:**
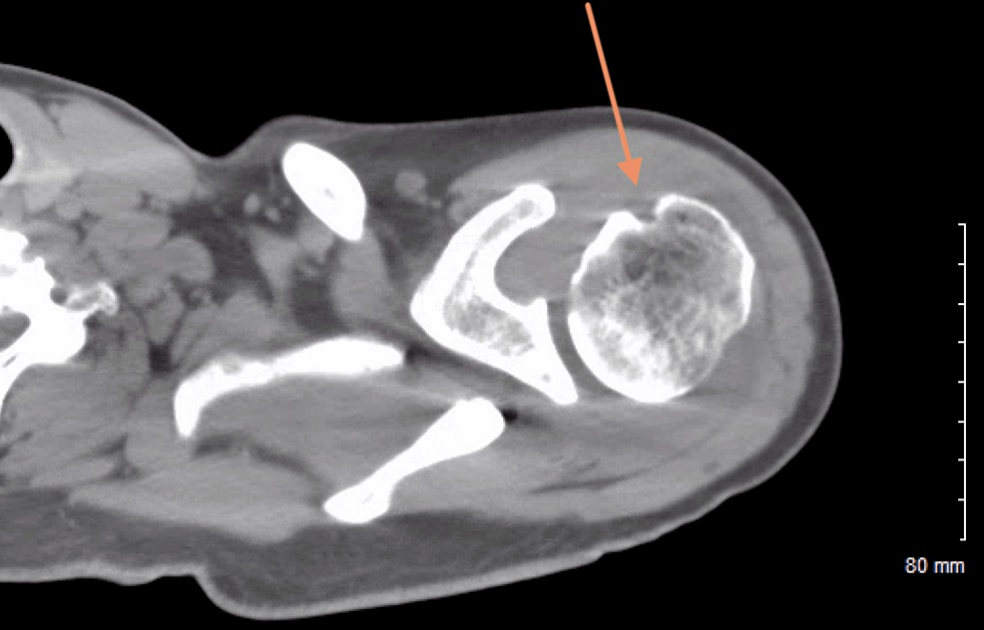
A computed tomography (CT) scan of the left shoulder Description: A CT scan with intravenous contrast of the patient’s left shoulder, demonstrating erosion at the humeral head (orange arrow) with surrounding soft tissue swelling

On day six of hospitalization, the patient reported increasing left shoulder pain that restricted his sleep. This pain was similar to a shoulder pain the patient complained of in the emergency room six months prior to this visit. The patient stated that it occurred after his dug tugged on the leash during one of their daily walks. An initial X-ray of the left shoulder revealed a focal erosion of the left humeral head with joint effusion, as seen in Figure 1. A magnetic resonance imaging (MRI) of the left shoulder without IV contrast was ordered and this further showed significant inflammation and edema in the joint spaces, as seen in Figure 2. Inflammatory markers revealed a sedimentation rate of over 140 mm/hr and high-sensitivity c-reactive protein (CRP) was above 8 mg/dL. A computed tomography (CT) scan of the left shoulder demonstrated erosion at the humeral head with soft tissue swelling with a possible septic joint, as seen in Figure 3. 

**Figure 4:**
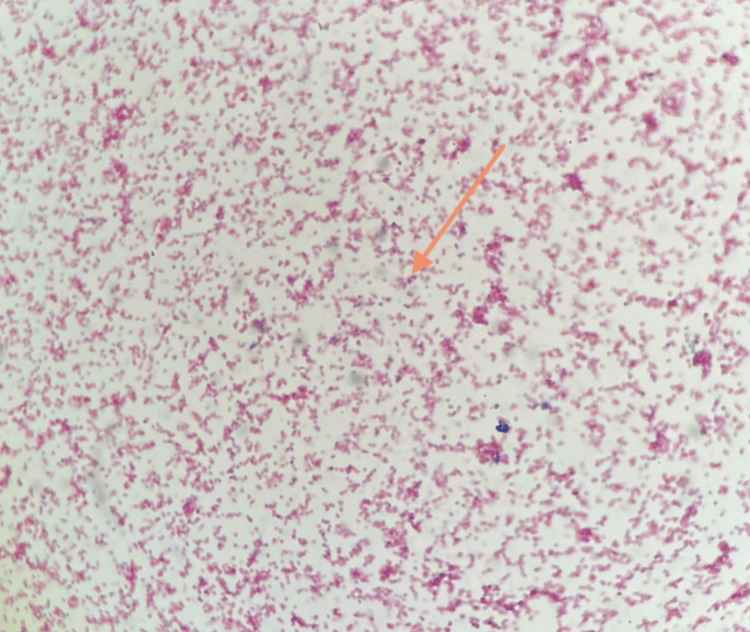
Pathology slide of the joint aspirate Description: Microbiology slide demonstrating Gram stain of N. gonorrhoeae specimen, a gram-negative diplococcus (orange arrow), obtained from the patient's joint aspirate

A joint aspiration of the left shoulder yielded 3 milliliters of cloudy serosanguinous fluid, which was sent for culture and cytology and can be seen in Figure 4. The following day, cultures from joint aspirate grew Gram-negative diplococci, beta-lactamase negative. The final wound culture report confirmed Neisseria gonorrhoeae.

The patient was started on ceftriaxone 1 g intravenous (IV) daily along with doxycycline 100 mg twice a day while chlamydial studies were pending. The patient had no urinary symptoms, lesions, or lymphadenopathy throughout hospitalization. Chlamydia trachomatis and N. gonorrhoeae polymerase chain reaction (PCR) amplifications were both negative. The doxycycline was stopped, and the patient had a peripherally inserted central catheter (PICC) line placed for long-term antibiotics. The patient was discharged with six weeks of ceftriaxone 2 g IV daily for joint and bone involvement with N. gonorrhoeae and negative blood cultures. The patient was safely discharged with outpatient weekly labs and close follow-up with Infectious Disease for disseminated gonococcal infection.

## Discussion

HIV is a retrovirus that selectively targets and infects CD4+ T-lymphocytes. This ultimately results in the death of the CD4+ T-lymphocytes and a resulting decrease in the host’s CD4-cell count. CD4+ T-lymphocytes normally play an important role in activating other immune cells, such as B-lymphocytes and cytotoxic T-cells, through the release of interleukins, e.g. IL-2, IL-4, IL-5, et cetera [4]. A reduction in CD4-cell count below 200 cells/mm^3^ of blood, known as AIDS, can therefore cause severe immunocompromise of the host, leaving the host vulnerable to opportunistic infections such as esophageal candidiasis, Pneumocystis jirovecii pneumonia, or cytomegalovirus retinitis [5].

Neisseria gonorrhoeae is a gram-negative diplococcus that usually colonizes mucous membranes of the oropharynx, genitalia, or anorectum. However, around 1% of cases may present as a disseminated disease with two distinct clinical presentations, arthritis-dermatitis syndrome or purulent gonococcal arthritis. In arthritis-dermatitis syndrome, patients may present with polyarthralgias, tenosynovitis, and dermatitis. In purulent gonococcal arthritis, patients may have abrupt inflammation in up to four joints (commonly knees, ankles, and wrist) with no skin manifestations and rarely tenosynovitis [6]. In this case, our patient’s gonococcal infection spread to the left shoulder joint, resulting in erosion of the left humeral head. It has been demonstrated that T-lymphocytes may produce complement proteins extra-hepatically following the activation of T-cell receptors and CD28 [7]. We believe the likely mechanism of dissemination in this patient was hematogenous spread following impaired recruitment of local complement mechanisms due to the patient’s low CD4 count (78 cells/mm^3^).

The proper workup for suspected gonococcal arthritis should begin with a proper history and physical. A patient presenting with asymmetric joint pain complicated by a history of sexually transmitted disease and lifestyle risk factors, such as multiple sexual partners, infrequent use of barrier contraceptives, female gender, IV drug use, or low socioeconomic status, but without any history of localized trauma, should raise suspicion for septic arthritides [8]. In fact, isolated monoarthritis should be assumed to be of infectious origin until proven otherwise [9]. A preliminary X-ray may be ordered to rule out mechanical causes, but imaging is rarely dispositive in monoarthridites, especially those absent a history of trauma [9]. As in this patient’s case, follow-up CT & MRI can help illuminate the etiology of possible soft-tissue pathologies, but the definitive diagnostic test is an image-guided aspiration of the joint with subsequent cultures of aspirate specimens [9]. Simultaneous culture to find the primary site of infection is high yield, as findings are positive in over 80% of cases [10]. Cultures from cervical, urethral, pharyngeal, and/or rectal sites may be indicated [10]. Blood cultures are usually negative [10]. Patients should also receive a full sexually transmitted infection workup for concomitant disease. In this patient, other possible differentials were also considered, including infectious arthritis, gout, secondary osteoarthritis, and avascular necrosis [11].

Treatment of the patient’s gonococcal arthritis consists of line placement and six weeks of intravenous ceftriaxone 2 g daily, with weekly monitoring of complete blood count, complete metabolic panel, erythrocyte sedimentation rate, and C-reactive protein to monitor for improvement of signs of inflammation and infection [8]. Initially, treatment to cover possible Chlamydial co-infection is indicated given the high likelihood of co-infection [8]. In our case, since nucleic acid amplification tests returned negative for Chlamydia, we suspended empiric doxycycline administration. Ceftriaxone is the preferred agent for gonococcal arthritis, as there is no gonococcal resistance to ceftriaxone currently demonstrated, even in the setting of increased resistance to fluoroquinolones and despite high rates of penicillin resistance globally [8].

Erosive monoarthritis has a number of differentials in radiography. Differentials considered include infectious arthritis, gout, secondary osteoarthritis, and avascular necrosis [11]. Rheumatoid arthritis was not considered as the patient did not present with polyarthritis [11]. The presence of joint space narrowing, effusion, and periarticular osteoporosis on radiography indicates an infectious origin until proven otherwise; this presentation is also not typical for gout, avascular necrosis, or osteoarthritis [11]. Poorly defined margins of bone destruction may be seen on shoulder radiographs. Gouty arthritis would present with well-defined margins, as would osteoarthritis secondary to joint trauma [11]. Avascular necrosis would present early without radiographic findings, which would develop into areas of patchy lucency and sclerosis not seen here [11]. Ultimately, the diagnosis of monoarthritis is dependent on image-guided aspiration with subsequent analysis [9]. Here, aspiration and culture of serosanguinous fluid from the joint space yielded the successful culture of N. gonorrhoeae, confirming the diagnosis of infectious gonococcal monoarthritis.

## Conclusions

This case highlights a rare complication and unusual location of disseminated Neisseria gonorrhoeae that can exist with AIDS or in immunocompromised patients. Disseminated gonorrhea is common in immunocompromised patients, thus we highlight the importance of working up the etiology of localized pain in a patient with AIDS. The workup includes obtaining appropriate imaging, which can include an X-ray, CT scan, and/or MRI. If imaging results are significant for either a fracture without reported local trauma to the site or erosions, we recommend obtaining an image-guided aspiration. If a disseminated infection is noticed, we recommend the appropriate treatment, which in our case was long-term IV antibiotics. If there is evidence of an infection, it is imperative to treat it with appropriate antibiotics and the right treatment course duration with close outpatient follow-up.
